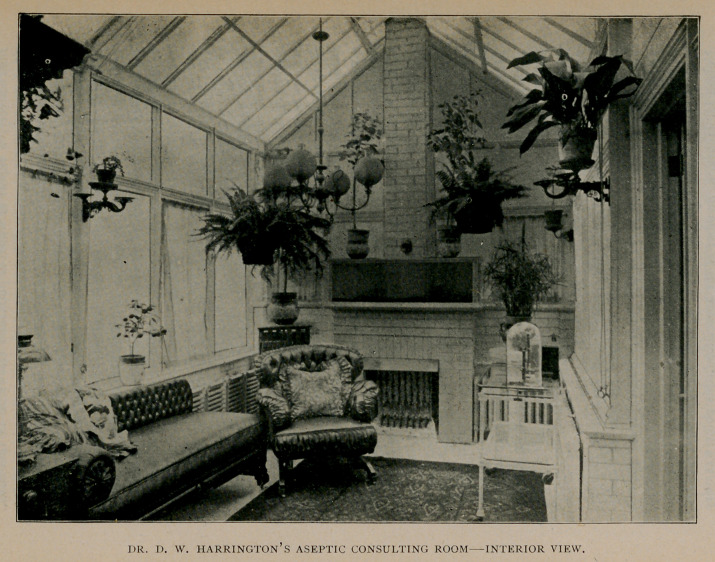# Medical Legislation at Albany

**Published:** 1900-04

**Authors:** 


**A Monthly Review of Medicine and Surgery.**

*EDITOR:*

WILLIAM WARREN POTTER, M. D.

All communications, whether of a literary or business nature, books for review and exchanges should be addressed to the editor · 284 FRANKLIN STREET, BUFFALO, N. Y.

VOL. XXXIX.—LV. APRIL, 1900. No. 9.

### MEDICAL LEGISLATION AT ALBANY.

THE number of bills relating directly or indirectly to medical affairs that the legislature is called upon to consider during the present session is surprising; especially so, when it is learned that very few of them are asked for by the medical profession as a body or by any considerable number of medical men as individuals. One would suppose that members of the legislature would get tired of the prevailing methods with reference to the introduction of bills and devise some way for relief.

With reference to medical bills the way to accomplish this is comparatively so simple, the only wonder is that it has not been adopted in the past. For example, there are at present three state medical societies representing three so-called systems of medicine, all of which have been chartered by the state, and all have been charged with certain specific duties relating to affairs that concern the state. To this number is about to be added a fourth—the New York State Medical Association—which has applied for a charter and undoubtedly will receive one. If the legislature would decline to receive a medical bill until it had received the recommendation of these societies; or, having received such a bill, would refer it to the chairmen of the legislation committees of the societies, it would relieve the senators and assemblymen of a lot of perplexity.

The several chairmen of the legislation committees of the four several state organisations could easily arrange for conferences from time to time in regard to such bills, and their approval affixed should be considered evidence of the public usefulness of a proposed law. On the contrary, their disapproval should be considered prima facie evidence that the bill is not necessary to the protection or advancement of the public health. In this way the medical profession would have ample notice of any legislation proposed for or against its interests as conservator of the public health of the state. This function—as guardian of public sanitation and to prevent disease—is the well-understood province of the profession of medicine; it is for this purpose the legislature granted charters to organise it into state and county societies, and it is for this purpose it created state medical examining boards. The latter, by elevating the standards of medical education and thus making it imperative upon the schools to turn out better doctors, has contributed not a little toward the fulfilment of the function above alluded to, a self-imposed duty that is always first and foremost in the minds of every well-bred and properly educated physician.

Having thus, by its own laws, erected the medical profession into corporate bodies for the purposes named, would it not be in the further extension of these purposes—namely, the prevention of disease and the promotion of health among the people, for the legislature to say in effect: We will consider no laws of a medical character until you have had an opportunity to examine them, and we will pass none such until your approval or disapproval has been stamped upon it through your duly constituted legislative committees? This would be a dignified way for the legislature to treat the medical profession and it would result in benefit to all concerned. In the foregoing we use the term "medical profession" to include all the different sects, so-called, that are recognised by law, and not in its restricted sense. What we desire is that in matters of medical legislation every legally qualified physician shall have a full voice and a free opportunity to exercise his rights.

Doctors are, for the most part, quite busy men, their services are personal in character and their duties and obligations to their patients are such that they can ill-afford to visit the capital repeatedly, for the purpose of persuading the legislature to hold off its hands. If such a plan as now suggested could be adopted it would benefit the whole commonwealth.

---

DR. W. W. KEEN, president of the American Medical Association, appreciating the importance of pathologic science, according to the *Journal* of the Association, has appointed unofficially a committee for the purpose of establishing a section on pathology in the Association. The members of this committee from New York are Drs. Roswell

Park, of Buffalo; Edward K. Dunham, of New York, and George Blumer, of Albany, all prominent members of the Medical Society of the State of New York or of its county branches. These are most excellent appointments, the men being skilled in the science of pathology. It is interesting to note that the president of the association thus honors the Medical Society of the State of New York in selecting his aides in scientific work. We do not quite understand, however, what the *Journal* means by *un*official appointments. We should say, rather, that the president in his official capacity had made the official appointment of a *provisional* committee, subject to the approval of the association. It is difficult to comprehend how such an act can be otherwise than official. At all events it is a good one, and reflects credit upon Dr. Keen.

---

In the February, 1900, issue of the Journal was published a description of an aseptic consulting office lately built in Buffalo, by Dr. D. W. Harrington, at his residence, 1430 Main Street. We take pleasure in presenting a picture of the interior of this unique and beautiful office on page 691 in this number. We have no doubt it will prove of interest to our readers in general, and especially to Dr. Harrington's many friends in this region. A visit to the office is well worth while, for though the picture conveys a good general impression of the room, it can only be thoroughly appreciated by personal inspection.

---

Dr. Francis E. Fronczak, in his report as physician to the penitentiary for 1899, directs attention to some evil practices that prevail with reference to commitments to that institution, as well as to the injustice of sentences in many instances. He says, among other things, that the mistake is frequently made of sending persons up for drunkenness who are really suffering from disease. These sufferers should be sent to the county hospital instead of the penitentiary, as a matter of course, and Dr. Fronczak is entitled to the thanks of the community for pointing out the evil. Moreover, as Dr. Fronczak says, drunkenness itself is not a crime, but a disease, and even such persons should not be punished as criminals; but the report recommends legislation to correct the evils named, and is a most creditable document in all its details.

---

Dr. Frederic S. Dennis, of New York, was the guest of honor at the meeting of the Surgical Section of the Buffalo Academy of Medicine, February 6, 1900. He presented for the consideration of the

DR. D. W. HARRINGTON'S ASEPTIC CONSULTING ROOM—INTERIOR VIEW.

section several absorbing surgical subjects and discussed them most instructively, a report of which is published elsewhere in this issue. He brought with him two assistants and two patients, the latter for clinical demonstration at the meeting. There, were, therefore, five persons in Dr. Dennis's party who came from New York for the purpose mentioned, for which Dr. Dennis assumed the entire expense, declining to be reimbursed by the academy. This is an unprecedented act of courtesy, as well as unselfish liberality, that deserves the highest commendation of every member of the medical profession in Buffalo.

---

Dr. Albert S. Ashmead, of New York, and Father L. W. Mulhaney, of Ohio, according to the *Tribune*, have drafted a national leper law for the suppression and prevention of leprosy in the United States. This bill, after calling attention to the evident spread of leprosy in every country where isolation is not practised, provides for a square mile to be set aside for a National Leper Home, either in Yellowstone Park or in some other dry climate antagonistic to the life of the leper bacillus. Buildings are provided for in the bill for use of the patients, and provision is made only for voluntary submission to the care of the government. The bill also makes provision, among other things, to prevent lepers from entering the United States in future.

---

The New York State Medical Association is asking for a charter at the hands of the present legislature, with a prospect that it will be granted. When we recall the somewhat grandiloquent manner with which it took itself out of the Medical Society of the State of New York a few years ago, asserting that it would have none of its politics, that it did not want legal recognition, but only to be let alone to discuss questions of science and ethics, it must be confessed the change of front is somewhat abrupt if not surprising.

However, viewed by itself the question is of very little interest, but when the motive is found to be the declared purpose of "assisting in the work of enforcement of the general medical laws" there is ample reason why the Medical Society of the State of New York should oppose the measure. At a recent hearing on the bill before the Assembly committee on Public Health, presided over by Dr. Nelson H. Henry, a member of the association, this point was very clearly made. Dr. E. D. Ferguson, the president of the State Association, who appeared for the bill, promptly denied that it was the purpose of the Association in asking for this charter to change the present practice with reference to the appointment of the State Medical Examining Boards, or to interfere with them in any manner. He said, moreover, that it was essentially a matter of sentiment with the Association in desiring a charter. Nothing could have been clearer than his expressed denial of any desire to obtain a division of the honors and responsibilities relating to the state examination for license to practise medicine.

It may be that this declaration on the part of the proponents of the bill, had quite as much to do with influencing the committee to favorable action as anything that was said. It is difficult to see how a charter could be denied to any respectable body of men seeking one, and especially when any attempt to injure another similar corporative body is disclaimed, but it would be well for the Medical Society of the State of New York to see that the bill is properly amended to include this disclaimer.

---

THE health of Paris is reported as unusually good for this season of the year. The sanitary reports of the Paris Board of Health, lately compiled, show that notwithstanding the prevailing influenza epidemic and a slight increase of smallpox—which is just now unusually frequent at Marseilles—the sanitary condition of Paris is more favorable than has been noted in the corresponding season for some years. It had been expected that the tunnelling and open way excavations for the Metropolitan Railway and the general tearing up of the streets made necessary by the new tramway system and by the works connected with the Exhibition, would lead to increased mortality through typhoid and malarial or infectious fevers. An examination of the official figures shows that these apprehensions were groundless; and, further, that typhoid fever is under satisfactory control, with the prospect of a considerable reduction in the death-rate the coming season. This will be good news for those who contemplate visiting the Paris Exposition during the coming summer.

---

THE Loud bill (H. R. 6071) is being considered again in Congress, and this time with prospects of passing unless strenuous efforts are made against it. Mr. Loud makes the seductive plea of economy—always very fetching with congressmen—in support of his measure. He says the postal service costs the government $45,000,000 now, and his bill reduces the deficit some $20,000,000. What the people want is an efficient, rapid, and frequent postal service, for which they are ready and willing to pay the bill. This measure proposed by Mr. Loud strikes a severe blow at medical journalism and ought not to pass in its present form. Every physician is interested, for if it passes his periodical medical literature will advance in price. Letters addressed to members of congress asking them to oppose the bill would have influence in preventing this measure, which Senator Depew declares is very unjust, from becoming a law.

---

VIOLATORS of the medical laws are not getting on well in Buffalo. Mrs. F. A. Andrews, of Clifton Springs, who was arrested recently on a charge of practising medicine without a license, was held for the grand jury by Police Justice Murphy. Hon. Tracy C. Becker appeared for the Medical Society of the County of Erie and conducted the prosecution. The charge against Mrs. Andrews is that she attempted to remove a birth-mark from the face of Clifford Kendall, the five-year old son of Mrs. Elsie Kendall, of No. 88 Southampton Street, and that she added to his disfigurement. Her bail was fixed at $500.

---

## Figures and Tables

**Figure f1:**